# TLR4 and Insulin Resistance

**DOI:** 10.1155/2010/212563

**Published:** 2010-08-10

**Authors:** Jane J. Kim, Dorothy D. Sears

**Affiliations:** ^1^Department of Pediatrics, University of California, San Diego, CA 92093-0673, USA; ^2^Rady Children's Hospital, San Diego, CA 92093-0673, USA; ^3^Department of Medicine, University of California, San Diego, 9500 Gilman Drive, La Jolla, CA 92093-0673, USA

## Abstract

Chronic inflammation is a key feature of insulin resistance and obesity. Toll-Like Receptor 4 (TLR4), involved in modulating innate immunity, is an important mediator of insulin resistance and its comorbidities. TLR4 contributes to the development of insulin resistance and inflammation through its activation by elevated exogenous ligands (e.g., dietary fatty acids and enteric lipopolysaccharide) and endogenous ligands (e.g., free fatty acids) which are elevated in obese states. TLR4, expressed in insulin target tissues, activates proinflammatory kinases JNK, IKK, and p38 that impair insulin signal transduction directly through inhibitory phosphorylation of insulin receptor substrate (IRS) on serine residues. TLR4 activation also leads to increased transcription of pro-inflammatory genes, resulting in elevation of cytokine, chemokine, reactive oxygen species, and eicosanoid levels that promote further insulin-desensitization within the target cell itself and in other cells via paracrine and systemic effects. Increased understanding of cell type-specific TLR4-mediated effects on insulin action present the opportunity and challenge of developing related therapeutic approaches for improving insulin sensitivity while preserving innate immunity.

## 1. Introduction

### 1.1. Insulin Resistance

Insulin resistance is a primary defect leading to and a characteristic feature of type 2 diabetes [1, 2]. The state of insulin resistance leads to increased insulin secretion by pancreatic *β*-cells and compensatory hyperinsulinemia. As long as compensatory hyperinsulinemia is sufficient to overcome the insulin resistance, fasting glycemia and glucose tolerance remain relatively normal. In patients destined to develop type 2 diabetes, *β*-cell compensation efficiency declines and relative insulin insufficiency develops leading to impaired glucose tolerance and eventually frank type 2 diabetes. Although there is still some debate as to whether the insulin resistance or the *β*-cell defect comes first, most epidemiologic studies indicate that in the early, pre-diabetic state, insulin resistance is the initiating abnormality.

Type 2 diabetes only develops in insulin resistant patients with a concomitant *β*-cell defect. As such, many subject groups with insulin resistance who do not have diabetes. These include patients with simple obesity, polycystic ovarian syndrome, and advanced age. There are a number of other abnormalities associated with insulin resistance that are included in the state of metabolic syndrome. Patients with metabolic syndrome are insulin resistant, hyperinsulinemic, and dyslipidemic (usually elevated triglyceride and decreased HDL levels) and frequently also have hypertension, nonalcoholic fatty liver disease, albuminuria, and increased plasminogen activator inhibitor 1 (PAI-1) levels. Epidemiologic evidence demonstrates that patients with metabolic syndrome have a high likelihood of developing type 2 diabetes and cardiovascular disease. Thus, while it is well established that treatment of insulin resistance has beneficial effects in patients with type 2 diabetes, it is becoming increasingly clear that enhanced insulin sensitivity is also therapeutically important in nondiabetic individuals with insulin resistance.

### 1.2. TLR4 Activity and Insulin Resistance-Associated Inflammation

**Figure 1:**
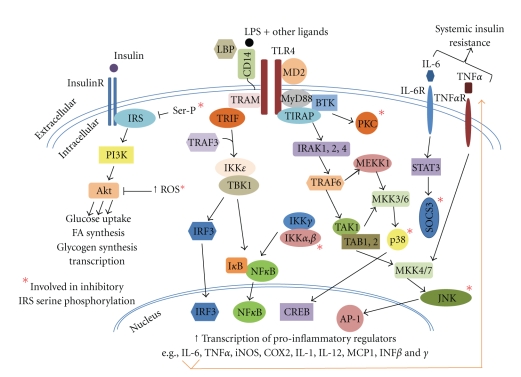
Schematic of TLR4 signaling cascades. Activation of TLR4 signal transduction through MyD88/TIRAP and TRAM/TRIF pathways leads to activation of innate immune responses and inhibition of insulin signal transduction, primarily through IRS serine phosphorylation. Additional cellular responses to TLR4 activation not shown include activation of NADPH oxidase, cytoskeletal rearrangement, and internalization of TLR4 complexes to endosomal compartments.

**Figure 2:**
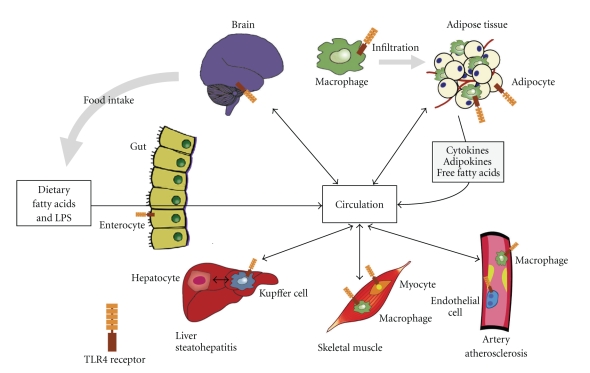
Distribution of TLR4 expression in the integrated organ/tissue systems that modulate energy homeostasis and insulin sensitivity. Schematic representation of the cross-talk between tissues. Adipose tissue shown in the macrophage-enriched, inflamed state.

Activation of the pro-inflammatory pathway has been described in a variety of insulin resistant states [3–6]. Chronic inflammation inhibits insulin sensitivity through the activation of signaling pathways that directly interfere with the normal function of key components of the insulin signaling pathway [5, 6]. Inflammation impairs insulin sensitivity in part via the activation of the Toll-Like Receptor (TLR) family of pattern recognition receptors, specifically TLR2 and TLR4. For the purposes of this review, we will focus on the role of TLR4 in insulin resistance. TLR4 is a cell surface receptor that generates innate immune responses to pathogens by inducing signaling cascades of kinase and transcription factor activation (Figure 1). These cascades lead to the generation of pro-inflammatory cytokines, chemokines, eicosanoids, and reactive oxygen species (ROS), all effectors of innate immunity. Notably, TLR4 is expressed in many cell components of insulin target tissues, including liver, adipose tissue, skeletal muscle, vasculature, pancreatic *β* cells, and brain (Figure 2). Thus, activation of TLR4 can dampen insulin action directly, through activation of pro-inflammatory kinases and ROS, and indirectly, via activation of cytokine signaling cascades and systemic release of pro-inflammatory, insulin-desensitizing factors (Figure 1).

Lipopolysaccharide (LPS) and its endotoxic moiety Lipid A are potent agonists of TLR4. LPS is an outer membrane component of gram-negative bacteria and is composed of oligosaccharides and acylated saturated fatty acids (SFA). Free SFA are also reported to bind and activate TLR4. However, there are conflicting interpretations of these data which are discussed in more detail below. Endogenous activators of TLR4 include S100A8/S100A9 (calprotectin) [7], high-mobility group 1 (HMBG1) [8], fibronectin [9], and minimally modified low-density lipoprotein (mmLDL) [10]. LPS binding protein (LBP), CD14 and MD-2 serve as TLR4 accessory proteins that facilitate ligand delivery in the circulation and receptor binding. Two signaling pathways are initiated by TLR4 activation (Figure 1). One, modulated by MyD88 and TIRAP, activates IKK, p38, JNK, CREB, AP2, and NF*κ*B and leads to the induction of pro-inflammatory genes. The other pathway, modulated by TRAM and TRIF, requires internalization of TLR4 (not depicted in Figure 1), activates IKK, NF*κ*B, and IRF3, and leads to induction of type 1 interferon genes. Transcriptional activation by these pathways induces robust expression of thousands of genes, depending on the cell type, that propagate the defense mechanisms of innate immunity. These signaling cascades induce feed forward signaling cascades (e.g., via IL-6 and TNF*α* receptor activation) and negative feedback loops (e.g., via transcriptional activation of the I*κ*B gene) [3]. In addition, TLR4-activated pathways inhibit the components of other signaling systems, for example, insulin signaling via IRS1 serine phosphorylation (Figure 1).

### 1.3. Regulation of TLR4

TLR4 expression and signaling are regulated by a variety of inputs, explained briefly here. Adiponectin impairs LPS activation of TLR4 signaling pathways in hepatic Kupffer cells and macrophages through mechanisms involving AMPK, IL-10, and heme oxygenase-1 [11–15]. AMPK also impairs LPS-induced I*κ*B degradation and CREB activation in macrophages [16]. Activated nuclear receptor transcription factors glucocorticoid receptor (GR), peroxisome proliferators-activated receptor gamma (PPAR*γ*), and liver X receptor alpha (LXR*α*) each transrepress TLR4-activated gene transcription and impair TLR4-mediated inflammation [17]. PPAR*γ* activation also inhibits expression of TLR4 [18, 19] and, conversely, TLR4 activation inhibits expression of PPAR*γ* [20]. Sex hormones can also affect TLR4 expression. Progesterone impairs LPS/TLR4 signaling efficacy via GR and progesterone receptor [21, 22]. Estrogen treatment of ovariectomized mice increases cell surface localization of TLR4 but does not change total cellular protein levels [23]. Testosterone downregulates TLR4 expression in macrophages both *in vitro* and *in vivo *[24]. Long-chain polyunsaturated omega-3 fatty acids (*ω*-3 FA) including DHA and EPA are antagonists of TLR4 activation by LPS and SFA in humans and mice [25–27]. One mechanism by which *ω*-3 FA interfere with TLR4 signaling is by altering plasma membrane lipid raft composition and function. For example, *ω*-3 FA block the ability of LPS and SFA treatments to stimulate assembly of TLR4 homodimers and signaling component complexes within lipid rafts, preventing subsequent signal transduction [28]. Stearoyl CoA desaturase 1 (SCD1) deficient mice, although protected from high-fat diet-induced insulin resistance, accumulate macrophage plasma membrane SFA, exhibit greater susceptibility to atherosclerosis, and are hypersensitive to inflammatory stimuli including those signaling through TLR4 [29, 30]. Supplementation with *ω*-3 FA completely protects SCD1 deficient mice from diet-induced atherosclerosis and metabolic syndrome [31]. These SCD1 deficient phenotypes may, in part, relate to lipid raft compositional changes that alter TLR4 signaling efficiency. SFA are conducive to formation and function of lipid rafts, essential sites of particular signal transduction pathways including TLR4, but lipid raft function and signaling component assembly are disrupted by *ω*-3 FA [32–34].

### 1.4. Role of Saturated Fatty Acids and Gut-Generated LPS as TLR4 Ligands

Are saturated fatty acids actual ligands of TLR4? SFA activation of TLR4 is an attractive link between obesity, insulin resistance, and inflammation, as cellular exposure to SFA greatly increases in the obese state. SFA are acyl components of LPS, activate TLR4 *in vitro*, and bind directly to TLR4/MD2/LPS crystal structures, although in an orientation that would probably rely on their presentation in an acylated form [35, 36]. Recent studies document endotoxin contamination of experimental reagents (such as bovine serum albumin, BSA) which would generate false-positive experimental results regarding the TLR4 agonistic effects of SFA [37–39]. LPS contamination is pervasive and LPS levels can only be assayed indirectly [31, 40]. Nonetheless, many publications document activation of TLR4 via SFA and many of these include samples that control for possible endotoxin contamination, for example, studies wherein BSA-complexed SFA treatments activate TLR4 effects but BSA alone and BSA-complexed monounsaturated fatty acid treatments do not. Extensive literature suggests that high-fat diet-augmented postprandial endotoxemia is a possible mode by which dietary SFAs induce inflammation through TLR4 in diet-induced obesity (DIO) models.

Both high-fat diets and high-fructose diets influence enterobacterial bacterial production and circulating levels of LPS/endotoxin by altering gut flora growth and composition and gut permeability [41–44]. Dietary lipids facilitate LPS incorporation into chylomicrons [45] and TLR4 is responsible for phagocytosis of gram-negative bacteria by gut enterocytes [46], each contributing to postprandial endotoxemia. Insulin resistant DIO and genetically obese mice and type 2 diabetic humans [41, 46–48] all exhibit elevated plasma LPS levels and endotoxemia is correlated with insulin resistance and atherogenic markers. Thus, chronic elevation of circulating gut-generated LPS or “metabolic endotoxemia” [49] would result in sustained, systemic pro-inflammatory stimulation of TLR4. Interestingly, germ-free mice or mice treated with antibiotics specific for gram-negative bacteria do not acquire high-fat diet-induced insulin resistance or other associated metabolic abnormalities [50–52]. Genetically obese *ob/ob* mice treated with an LPS inhibitor or in a CD14 KO background have reduced inflammation and metabolic abnormalities compared to normal *ob/ob* mice [51, 52] which suggests that these *ob/ob* phenotypes are partly mediated by gut LPS and TLR4 signaling.

### 1.5. Mouse Model Overview

Several mouse model studies have demonstrated the importance of TLR4 and its signaling components in diet-induced insulin resistance, inflammation, and atherosclerosis. These studies include those conducted in whole body TLR4 knockout (KO) or loss-of-function mutations [26, 53–55], hematopoetic TLR4 KO [56], and whole body KOs of MyD88 [57] or CD14 [58, 59]. Some discrepancies in phenotypic reports with regard to food intake, weight gain, and adipose tissue macrophage accumulation will be discussed in the sections below. There are two nonsynonymous polymorphisms (SNPs) in the human TLR4 gene that result in changes in the TLR4 extracellular domain. These polymorphisms are reported to alter responsiveness to TLR4 activation and correlate with protection from atherosclerosis, CVD and the metabolic syndrome in some populations [60–62].

In the next sections, we will highlight tissue-specific effects of TLR4 activation and its role in insulin resistance. As many of the mouse models are whole body KOs of TLR4 signaling, it is unclear which exact cell type(s) mediate the phenotypes observed in these models. TLR4 expression in tissues, the mediate glucose homeostasis, and insulin sensitivity is shown in Figure 2 along with a schematic representation of the intra- and intertissue crosstalk that exists *in vivo*.

### 1.6. Adipose Tissue

Adipose tissue acts not only as a storage depot for excess calories, but also secretes large numbers of hormones, cytokines, and chemokines that influence energy homeostasis and metabolism (Figure 2). Adipose tissue consists of a variety of cell types, including adipocytes, immune cells (macrophages and lymphocytes), preadipocytes, and endothelial cells. Among these cell types, adipocytes and macrophages release cytokines and chemokines such as MCP-1, IL-1*β*, IL-6, and TNF-*α* [5] that promote inflammation. In addition, adipocytes are the unique source of hormones termed “adipokines" such as leptin [63, 64] and adiponectin [65], which can promote insulin sensitivity as well as resistin and retinol-binding protein 4 (RBP4), which can impair insulin sensitivity [6].

In states of chronic nutrient excess and obesity, insulin resistance manifests through several mechanisms such as increased free fatty acid (FFA) flux, ER stress, and microhypoxia in adipocytes, all of which are associated with increased inflammation [6]. In recent years, much interest has been focused on this association between obesity, chronic inflammation and insulin resistance. In 2003, two landmark studies showed bone-marrow derived macrophages invade adipose tissue in obese states [66, 67]. Recruited macrophages may initially remove dying cells and contribute to adipose tissue vascularization, but their activation towards an inflammatory phenotype soon results in cytokine production [68]. These macrophages contribute to the development of inflammation in adipose tissue and are believed to be a key contributor to insulin resistance [69]. This relationship between increased macrophage infiltrates and obesity has since been verified in human studies [70]. A growing body of literature is focused on the role of these adipose tissue macrophages (ATMs) in insulin resistance, characterizing their activation, recruitment, and function.

Attenuating the inflammatory signaling pathway by gene knockout experiments has been shown to reduce obesity-related insulin resistance in mice [69, 71–74]. For example, mice with myeloid cell-specific deletion of IKK*β* or JNK1 have significantly improved glucose tolerance and insulin sensitivity despite high-fat diet-induced obesity. Several lines of evidence point to the involvement of TLR signaling in this paradigm. For example, TLR4 expression is highly abundant in pro-inflammatory macrophages [75, 76] and differentiated adipocytes [77, 78]. Moreover, TLR4 expression in adipocytes increases with obesity [26] and it can be activated by LPS to induce NF*κ*B activation and cytokine production in both rodent and human tissues [79–81]. SFA acylated in the lipid A moiety of LPS is essential for the biological activity of LPS [82]. LPS and SFA have also been shown to attenuate insulin signaling in adipocytes with decreased phosphorylation of Akt and GSK3*β* [78].

Most intriguingly, TLRs in adipocytes [26, 83] and pro-inflammatory macrophages [76] may be directly activated by nutrients, particularly SFA. Although it is not yet clear if LPS contamination may contribute to any of these findings, dietary SFA appear to differentially modify the risk of developing many chronic inflammatory diseases in both human and animal studies [84]. In contrast, fish oil-derived *ω*-3 FA can mitigate the effect of LPS and free SFA-induced inflammation in macrophages [25] and adipocytes [26] and prevent high-fat diet-induced insulin resistance in rodents [85]. Taken together, this evidence suggests that elevated FFAs in obesity activate TLR signaling and impair insulin action, providing yet another link between obesity, inflammation, and insulin resistance.

Toll-like receptors in adipose tissue play a key role in initiating the inflammatory response, thereby promoting insulin resistance. Several studies to date have shown that disruption of the TLR4 gene in mice confers protection from obesity-induced inflammation and insulin resistance [26, 54, 86–88]. Several models of TLR4 deficiency have been studied in this context. Two LPS-resistant naturally occurring mouse strains have been identified with loss-of-function (C3H/HeJ mice) or deletion (C57BL/10ScN mice) mutations in the TLR4 gene [55, 81]. TLR4 null mice have also been generated using homologous recombination [80]. Body composition differs between mouse strains following high-fat feeding, with unaltered [88] or decreased [55, 86, 87] weight in C3H/HeJ and C57BL/10ScN mice, and unaltered or increased weight in *TLR4^−/−^* mice [26]. It is unknown whether this divergence results from differences in TLR4 mutations, mouse strains, or other factors. However, in both models, TLR4 signaling in macrophages and adipose tissue appears to regulate whole body glucose homeostasis via effects on adipose, muscle, and liver tissues.

Since body composition strongly influences insulin sensitivity, it is challenging to ascertain whether improved glucose metabolism results from altered TLR4 expression or decreased body weight. Therefore, models in which body weight is unaltered or increased are advantageous. There are two papers published to date showing increased adiposity following high-fat feeding in mice with disrupted TLR4 gene expression [26, 88]. In these studies, adipose inflammation was greatly attenuated with reduced expression of pro-inflammatory genes, despite their greater adiposity. TLR4 deletion also improved insulin sensitivity, with higher rates of glucose disposal into skeletal muscle and adipose tissue, and reduced inflammation and insulin resistance induced by either LPS or FFAs in isolated primary adipocytes [26, 88]. Moreover, while *in vivo* lipid infusion promotes insulin resistance and NF*κ*B activation in adipose tissue of control mice, similar effects were not observed in TLR4 knockout mice [26].

Additional work indicates that TLR4-mediated alterations in macrophage activity underlie the adipose-specific improvements in inflammation and insulin. Saturated fatty acids such as palmitate, lauric acid, and oleate fail to elicit TNF*α* production or IKK*β* degradation in elicited peritoneal macrophages from TLR4 mutant mice [26, 89]. Furthermore, mice with myeloid-specific ablation of TLR4, generated by transplanting bone marrow from *TLR4^−/−^* animals into wild-type mice, demonstrate significantly improved insulin sensitivity in adipose tissue with reduced ATMs and adipose pro-inflammatory gene expression [54]. Because these chimeric mice possess TLR4 deficiency only in bone marrow-derived cells, it is possible to determine the contribution of hematopoietic TLR4 to insulin sensitivity. In addition, body weight and adipose depot sizes are equal between chimeric and control mice, such that differences in adiposity are not a confounding factor in the comparison between groups. Similar conclusions were drawn from studies in mice lacking the TLR4/TLR2 coreceptor CD14. Adipose tissue from obese CD14 null mice had fewer macrophages, with reduced glucose intolerance despite increased body weight [59]. Interestingly, TLR4 expression in peripheral blood mononuclear cells is reduced in overweight individuals with metabolic syndrome that undergo weight loss [90]. Although our understanding of macrophage function is incomplete, it appears that their absence in adipose tissue confers protection against insulin resistance during nutrient overload.

### 1.7. Liver

The liver is comprised of heterogeneous cell types including hepatocytes, stellate cells, endothelial cells, and immune cells. Liver-resident macrophages called Kupffer cells make up 10% of cells in the liver and 80%–90% of all tissue macrophages in the body [91]. They are localized at sinusoids where they are in close contact with circulating factors (hormones, cytokines, lipids, danger signals, postprandial LPS, etc.). Kupffer cells, able to migrate and cross-talk with other cell types within the liver, are important mediators of liver inflammation and non-alcoholic fatty liver disease (NAFLD), reviewed in more detail within this volume [92]. TLR4 is expressed on Kupffer cells and other liver cell components and modulates liver pro-inflammatory activity induced by high-fat diet- and fructose-induced hepatic steatosis and insulin resistance in mouse models [26, 44, 54, 93], although a direct role for liver tissue TLR4 in these processes is unclear. In obese patients with nonalcoholic steatohepatitis (NASH), liver tissue levels of activated pro-inflammatory NF*κ*B and AP-1 were correlated with oxidative stress and insulin resistance [94]. Both whole body and myeloid-specific TLR4 signaling deficiency result in reduced lipid accumulation, inflammation, JNK and IRS1 serine phosphorylation, and insulin resistance in liver [26, 54, 59, 86–88]. Interestingly, acute LPS treatment inhibits hepatic glucose production *in vivo* and *in vitro* via a TLR4 signaling pathway and induces insulin resistance 48 hours post-treatment *in vivo*, suggesting possible cross-talk between TLR4 and insulin receptor signaling pathways in this system [95]. The hypoglycemic effects of acute LPS are PI3K- and TNF*α*-independent and additive when combined with insulin. There are several models of how steatosis could facilitate activation of TLR4 in Kupffer cells [93]. Increased lipid content and exposure can affect TLR4 signaling complex assembly, endosomal sorting, and signaling cascade flow by altering lipid raft composition and membrane fluidity. Liver hypertrophy and elevated lipid content can impair sinusoidal perfusion efficacy and constrict circulatory flow. Circulating leukocytes, stuck within the sinusoids, would be more likely to activate liver resident cells, including Kupffer cells. Liver tissue infiltration of circulating monocytes might also be facilitated in the inflamed, hypertrophic, and steatotic state. In the insulin resistant state, liver-expressed TLR4 would be exposed to elevated circulating ligands including adipose tissue FFA and, in obese/high-fat diet states, LPS and dietary SFA.

### 1.8. Muscle

Skeletal muscle is the primary site for insulin-stimulated glucose utilization, accounting for over 75% of this process under normal physiological conditions [96, 97]. Glucose disposal into muscle is markedly reduced in obese, hyperinsulinemic subjects [98], underscoring the importance of skeletal muscle in normal glucose homeostasis and in the development of insulin resistance. Putative mechanisms of insulin resistance in skeletal muscle include direct effects of intramyocellular FFA metabolites, paracrine effects of adipocytes, and macrophages (interspersed between muscle fiber bundles) in muscle tissue as well as endocrine effects from adipocytes and macrophages present in adipose tissue. As an example of the latter, the production of the pro-inflammatory cytokine TNF*α* from adipose tissue can impair insulin signaling in muscle through inhibitory serine phosphorylation of IRS-1 [99].

TLR4 has been shown to regulate substrate metabolism in muscle, favoring glucose oxidation rather than fatty acid oxidation in the absence of insulin [100]. Emerging data indicates that TLR signaling may also underlie the development of chronic inflammation and insulin resistance in skeletal muscle. Skeletal muscle cells and intact whole muscle express multiple TLRs, including TLR2 and TLR4 [101], that are responsive to LPS [102]. Moreover, skeletal muscle TLR4 gene and protein expression are significantly elevated in muscle from obese subjects with type 2 diabetes [103]. In these individuals, TLR4 protein expression in muscle correlates with the severity of insulin resistance. TLR4 contributes to skeletal muscle metabolism. Activation of TLR4 has also been shown to regulate substrate utilization in muscle, favoring glucose oxidation rather than fatty acid oxidation in the basal state.

Much of the current data implicates saturated FFAs as ligands for TLRs in muscle tissue. For example, in human myotubes and in muscle from lean human subjects, acute palmitate treatment induces robust NF*κ*B-activation via TLR4 [103]. In C2C12 mouse myotubes, palmitate has been also shown to activate NF*κ*B, as well as JNK1/2 and novel PKC pro-inflammatory pathways via TLR2 [104]. Rodent studies also show that disrupted expression of TLR4 protects against saturated fatty acid-induced insulin resistance in muscle resulting in improved insulin-stimulated glucose uptake, improved IRS1 tyrosine phosphorylation, reduced IRS1 serine phosphorylation, and decreased JNK1 phosphorylation in TLR mutant mice [26, 87]. More recent work indicates that conditioned media from macrophages treated with palmitate, but not LPS, can impair glucose uptake in muscle cells, suggesting that saturated fatty acids mediate their effects on muscle via macrophage cells [68].

### 1.9. Other Tissues

#### 1.9.1. Brain

 In recent years, much interest has been focused on the role of the brain in regulating glucose metabolism. Several investigators have identified the hypothalamus and mesolimbic area as important sites in the regulation of food intake, energy expenditure, peripheral insulin resistance, and pancreatic *β*-cell function. For example, neural influences on the liver and muscle directly influence glucose output and uptake in these tissues [105]. Inflammatory signaling pathways in adipose tissue have been shown to regulate energy balance by increasing thermogenesis [106]. Studies in TLR4 mutant mice suggest that TLR signaling in the CNS may also contribute to obesity phenotypes by affecting nutrient intake. For example, TLR4 null females demonstrate increased adiposity secondary to increased food intake on both normal chow and high-fat diets [26]. In addition, a study of C3H/HeJ mice showed decreased food intake when fed ad libitum, and developed obesity when pair-fed with control mice [88]. It is difficult to ascertain direct effects of TLR signaling in the brain since TLR4 expression is disrupted in the whole body in both models. However, high-fat diet has been shown to increase JNK1 and NF*κ*B activation in the hypothalamus with impaired insulin signaling and apoptosis that may result in dysregulated feeding control [107]. TLR4 in the hypothalamus may activate pro-inflammatory pathways that contribute to the development of insulin and leptin resistance. For example, mice deficient for the TLR adaptor molecule MyD88 in the CNS are protected from hypothalamic inflammation and leptin resistance induced by acute central application of palmitate as well as from impairment of peripheral glucose metabolism induced by either centrally administered palmitate or by HFD [108]. However, the role of TLR4 is still unclear as some work suggests that TLR4 may in fact play dual roles in the hypothalamus by both activating pro-inflammatory pathways and restraining apoptosis [109].

#### 1.9.2. Pancreatic Islet

 Several TLRs (TLR2, 3 and 4) are highly expressed in human and rodent islets [110]. Although types 1 and 2 diabetes differ in etiology, *β*-cell destruction eventually occurs in both cases, leading to clinical manifestations of absolute or relative insulin deficiency. Islet inflammation is a well-established observation in autoimmune-mediated type 1 diabetes. However, it has been more recently described in type 2 diabetes and is thought to be secondary to toxicity induced by high glucose, FFAs, cytokine signaling, or ER stress. TLR signaling in *β*-cells has been primarily implicated in autoimmune type 1 diabetes [111–114]. Nevertheless, recent studies also implicate TLR2/4 and MyD88 in insufficient *β*-cell compensation in type 2 diabetes. For example, the chemokine CXCL10 is highly expressed in islets isolated from individuals with type 2 diabetes and has been shown to impair insulin secretion and promote *β*-cell apoptosis via TLR4 [115]. In addition, TLR activation by FFAs can induce the expression of proinflammatory cytokines in purified mouse and human islets [116], suggesting that the dyslipidemia associated with insulin resistance promotes islet inflammation.

#### 1.9.3. Artery and Endothelial Cells

 TLR4 activity impairs endothelial cell function and contributes to atherosclerosis. TLR4 is required for LPS-stimulated NF*κ*B activation in endothelial cells [117]. Kim et al. report that mice with whole body deletion of TLR4 were protected against high-fat diet-induced vascular inflammation (aorta) *in vivo* [53]. The mice were also protected from high-fat diet-induced insulin resistance while exhibiting the same body weight, adiposity, and plasma insulin and FFA levels as wild-type controls. These authors also demonstrated that the SFA palmitate stimulated TLR4-dependent IKK and NF*κ*B activation and impairment of insulin signaling and NO production in wild type aortic explants and cultured human endothelial cells. Whole body disruption of TLR4 signaling prevents atherosclerosis in proatherogenic genetic mouse models [57, 58]. Interestingly, in hematopoetic cell TLR4 KO/whole body LDLR KO agouti mice, only females fed normal chow plus cholesterol diet exhibited less ATM, inflammation, and atherosclerotic lesion size than TLR4 wild-type controls [56]. In these studies, metabolic and atherogenic sequelae were the same in hematopoetic cell TLR4 KO versus wild type females fed three different high-fat diet plus cholesterol formulae. The authors state that no significant phenotypic differences were observed between the male genotypes. Direct action of TLR4 signaling in vascular inflammation and atherosclerosis in vivo is unclear as the phenotypes described above could be mediated by TLR4 indirectly via reduced inflammation elsewhere (e.g., adipose tissue).

#### 1.9.4. Sex-Specific Dimorphism

Numerous observations in the literature reveal sexual dimorphism in the regulation of TLR4 and TLR4 signaling. Sex hormones differentially regulate TLR4 localization, gene expression, and sensitivity, described above. Males and females exhibit dramatic differences in their immune responses and susceptibility to sepsis [118]. Innate immunity modulation and insulin resistance are observed in pregnancy during which estrogen and progesterone levels are dramatically elevated [119–121]. Two of the TLR4 knockout mouse studies described above report TLR4-dependent phenotypic effects in females only [26, 56]. Sex differences in acute lipid infusion-induced insulin resistance have been reported in both human [122] and rat [123] studies where only males become insulin resistant during lipid infusion. Future studies should provide more clarity about the mechanisms by which TLR4 signaling is modulated by sex hormones.

## 2. Conclusion

TLR4 activation promotes insulin resistance. Given the wide-spread expression pattern of TLR4 in tissues and cell types that modulate energy homeostasis and insulin action (Figure 2), the direct, cell type-specific relationship between TLR4 activation and insulin resistance is unclear. TLR4 plays an important insulin-desensitizing role in myeloid-derived cells and future studies of nonmyeloid, cell type-specific transgenic and knockout models are of great interest. Our increasing understanding of TLR4 and insulin resistance will facilitate the design of novel therapeutic approaches that can derail the negative metabolic effects of TLR4 activation from the important functions of innate immunity.
